# Directions

**DOI:** 10.1155/S106474499700001X

**Published:** 1997

**Authors:** Sebastian Faro


Infectious Diseases in Obstetrics and Gynecology 5:1 (1997)
© 1997 Wiley-Liss, Inc.

### Directions

The journal *Infectious Diseases in Obstetrics and Gynecology* has traveled a significant distance thus far in its short existence. The journal is assuming a prominent role in representing the Infectious Disease Society for Obstetrics and Gynecology. Recently the Society chose the journal to be its official publication. This is an important step in our evolution, since the journal has also been selected as the official publication of the United States section of the International Disease Society for Obstetrics and Gynecology.

The development of new sections within the journal such as Images and the Antimicrobial Symposium have widened the breadth of the journal's appeal to practitioners as well as residents. In this issue we debut the section "Challenges in Infectious Diseases." We know that our readers face challenges daily and we welcome those submissions to the journal. We hope that this section will become a reference readers check when they are faced with these types of problems.

*Infectious Diseases in Obstetrics and Gynecology* has also increased its publication of manuscripts from around the world. Thus, we have truly become an international vehicle for communication to all physicians involved in the treatment and research of infectious diseases of women. The journal's component parts currently offer articles of interest to the broad range of individuals dedicated to providing health care to women.

We continue to be committed to providing not only the standard type of articles, but to be innovative in the delivery of information. The future is bright for the journal, and we continue to seek input from the readers regarding ways to help serve the subscribers and the field of infectious diseases.

Through publication of the manuscripts of material presented at the International Chlamydia meeting, IDSOG, and USIIDSOG, we strive to provide current information to the readership. We will continue our efforts to consolidate and present information pertinent to infectious diseases in women. The next goal of the journal is to obtain listing in *Index Medicus* and we will work very hard to achieve this goal.

*Sebastian Faro, MD, PhD*
*Editor-in-Chief*

